# Perceptions of risk of SARS-CoV-2 transmission in social and educational activities by infectious diseases and general pediatric healthcare providers, a pre-vaccine risk perception cross-sectional survey

**DOI:** 10.1371/journal.pone.0263767

**Published:** 2022-02-11

**Authors:** Andrew B. Janowski, Philip M. Polgreen, Susan E. Beekmann, Jason G. Newland

**Affiliations:** 1 Department of Pediatrics, Washington University School of Medicine, St. Louis, Missouri, United States of America; 2 Department of Internal Medicine, The University of Iowa, Iowa City, Iowa, United States of America; National Institutes of Health, UNITED STATES

## Abstract

**Background:**

The perception of the transmission risks of SARS-CoV-2 in social and educational settings by US healthcare providers have not been previously quantified.

**Methods:**

Respondents completed an online survey between September and October 2020 to estimate the risk of SARS-CoV-2 transmission on a scale of 0–10 for different social and educational activities prior to the availability of the SARS-CoV-2 vaccines. Demographic information and experiences during the pandemic were also collected. The risk assessment was emailed to three listservs of healthcare providers, including national listservs of pediatric (PID) and adult infectious diseases (AID) providers, and a listserv of general pediatric practitioners in the St Louis, USA metropolitan area.

**Results:**

Respondents identified the highest risk of SARS-CoV-2 transmission in spending time in a bar, eating at a restaurant, and attending an indoor sporting event. In the school setting, lower risk was identified in elementary and daycare students compared to high school or university-level students. Comparatively, the risk of transmission to students and teachers was lower than the identified high-risk social activities. Factors increasing risk perception in social activities included the absence of children in the respondent’s household and female gender. For the school setting, AID providers perceived greater risk compared to PID providers or pediatric practitioners.

**Conclusions:**

Respondents identified high risk activities that were associated with a high density of participants in an indoor space where masks are removed for eating and drinking. Differences were apparent in the school setting where pediatric providers perceived lower risks when compared to adult providers.

## Introduction

In 2019, the severe acute respiratory syndrome coronavirus 2 (SARS-CoV-2) emerged and subsequently caused a global pandemic of coronavirus disease 2019 (COVID-19) [1]. This virus caused significant disruption to healthcare systems and subsequent temporary closures of schools and businesses [1]. As understanding of the biology of the virus has emerged over time, there is considerable interest in how SARS-CoV-2 is transmitted, factors that potentiate the risk in social activities, and methods which reduce the risk of transmission [2, 3]. Healthcare providers have been rated as the most trusted source of information during the pandemic and have been a frequent source of education for the public regarding the risks of SARS-CoV-2 transmission [4, 5]. However, very few epidemiological studies exist that attempt to quantify the risk for specific social activities and in the school setting prior to the wide-spread availability of SARS-CoV-2 vaccines [6–8]. Various educational news stories and materials have been generated, but many of these risk assessments are based on the views of a small number of experts which could be prone to bias [9–14]. Furthermore, the factors that influence how healthcare providers perceive the risk of transmission have not been described. Experiences during the pandemic and personal factors could influence these perceptions as identified in other populations [15–21] but it is not clear if these factors also affect the perceptions of US medical providers. In order to better understand the perceptions of risk by healthcare providers and the factors that influence these scores, we queried pediatric and adult infectious diseases specialists and general pediatricians regarding the risks of transmission of SARS-CoV-2 while participating in common social activities and the perceived risk in the school setting to students and teachers.

## Materials and methods

The Washington University in St Louis Institutional Review Board approved this study (IRB# 202009013). We developed a web-based form to assess risk (https://ein.idsociety.org/surveys/survey/131/) that was distributed to members of three different email listservs from September 17^th^, 2020 to October 18^th^, 2020: 1) the Infectious Disease Society of America (IDSA) Emerging Infections Network (EIN) listserv that includes both adult and pediatric infectious diseases physicians [22], 2) Pediatric ID listserv managed by Washington University School of Medicine and the SHARPS Collaborative, and 3) the Washington University Pediatric and Adolescent Ambulatory Research Consortium (WUPAARC) listserv managed by Washington University School of Medicine. Members of these listservs included nurses, nurse practitioners, pharmacists, epidemiologists, general pediatricians, pediatric infectious diseases physicians, and adult infectious diseases physicians. A total of approximately 3,700 health care providers are included in these three listservs. Follow-up reminder emails were sent one and two weeks after the initial email. For an estimation of sample size for a power calculation, we used G*Power 3.1.9.4 [23]. Using a moderate effect size of 0.15 for a linear regression model with an α of 0.05, power of 0.95, and 7 predictors, we obtained a minimum total sample size of 153 subjects.

Respondents were asked to estimate the generalized risk of transmission of SARS-CoV-2 using a 0 to 10 point scale (10 being the highest risk) for 14 different social activities prior to the wide-spread availability of SARS-CoV-2 vaccines and emergence of the SARS-CoV-2 delta variant. Activities were described in both the context of universal mask wearing and social distancing and also in the context of no usage of masks or implementation of social distancing. These activities were assessed under the assumption that there is local transmission of COVID-19 cases, but local hospitals are not at capacity and the seven-day rolling average of the daily new COVID-19 cases has plateaued. Three non-social activities were assessed not using the with or without masks/social distancing approach, as respondents were asked to assess the risk from working from home, providing medical care to COVID-19 patients without any personal protective equipment (PPE), and providing medical care to a COVID-19 patient with full PPE.

For the educational setting, respondents estimated the generalized risk to students and teachers at the college, high school, elementary, and daycare (age <5 years) levels, using the same 0 to 10-point scale. The risk in schools was assessed under the following assumptions: 1) each educational center is following the American Academy of Pediatrics and CDC recommendations for school/daycare reopening with appropriate methods for cleaning, ventilation, social distancing, wearing masks, physical barriers where possible, cohorting, and methods in place for identifying potentially sick children [24, 25]; 2) All classroom situations are limited to <30 attendees; and 3) For educators, assume the person is age 40 without any past medical history or risk factors for severe COVID-19 disease 4) all situations were assessed prior to wide-spread availability of the SARS-CoV-2 vaccines and emergence of the SARS-CoV-2 delta variant. Demographic information was collected on respondents including gender, job title, specialty, age group for which they provide primary care, years since completing terminal degree or training, the presence of children (age < 18 years) in their household, practice location, whether they have provided care to COVID-19 patients, whether they have experienced a surge in COVID-19 patients that has exceeded hospital capacity, whether a mask mandate has been enacted in their practice area, estimation of the frequency of mask wearing in public, and an estimation of the primary approach most schools in their area are taking to control the pandemic.

Data were analyzed using SPSSv27 (IBM) and Prism v8.1 (Graphpad). Summary statistics were calculated for each demographic variable. A Friedman’s test was conducted to identify significant differences in the risk assessment of the different activities and school settings, and a Dunn-Bonferroni test with a Bonferroni correction was used for post-hoc testing to control for multiple comparisons. To determine the factors that influence the 14 common activities, a generalized linear model was developed. The outcome of this model was each participant’s average risk score across those activities with and without masking/social distancing with covariates derived from the demographic information and experiences of the respondents during the pandemic. Model diagnostics were used to confirm the appropriateness of the analysis including Levene’s test of homogeneity of variance, and assessment of outliers using Cook’s Distance and standardized residuals. Two outliers were removed from the final analysis which improved the homogeneity of variance value and shifted the Kolmogorov-Smirnov test to a p-value of >0.05 but did not significantly change the estimated marginal means of individual factors. For the assessment of risk to students and teachers, the same approach was used. An average risk score was determined for each respondent and used in a generalized linear model. Model diagnostics were analyzed and no significant outliers were identified, demonstrating the initial model to be appropriate. A Friedman’s test with post-hoc testing via the Dunn-Bonferroni test were used to determine which group of students and teachers were determined to have the highest and lowest perceived risk of SARS-CoV-2 transmission. To determine factors that influenced the scores for each group of questions about students and teachers, ordinal regressions were used and adjusted odds ratios calculated. For all tests, p-values ≤ 0.05 was considered significant.

## Results and discussion

We received responses from a total of 403 individuals. Demographic data of the cohort is displayed in Table 1, and respondents’ experiences during the pandemic are presented in Table 2.

**Table 1:** Demographic data of 403 respondents.

		N (%)
Gender	Female	229 (56.8)
	Male	162 (40.2)
	Prefer not to say/no answer	12 (3)
Specialty	Adult infectious diseases	146 (36.2)
	Pediatric infectious diseases	161 (40)
	General pediatrics	83 (20.6)
	Combined internal medicine-pediatrics	4 (0.9)
	Internal medicine	2 (0.5)
	Family medicine	4 (1)
	No answer	3 (0.7)
Time in specialty	< 5 years	61 (15.1)
	5–14 years	139 (34.5)
	15–24 years	97 (24.1)
	>25 years	102 (25.3)
	No answer	4 (1)
Role	Physician (MD or DO)	368 (91.3)
	Advanced Practice Provider (NP or PA)	10 (2.5)
	Pharmacist	11 (2.7)
	RN	1 (0.2)
	Epidemiologist	4 (1)
	Other	3 (0.7)
	No answer	6 (1.5)
Practice region	West	68 (16.9)
	Midwest	184 (45.7)
	South	74 (18.4)
	Northeast	66 (16.4)
	Puerto Rico	1 (0.2)
	Canada	2 (0.5)
	Other	4 (1)
	No answer	4 (1)
Do you have children (<18 years of age) in your household?	Yes	226 (56.1)
	No	172 (42.7)
	No answer	5 (1.2)

Abbreviations: MD doctor of medicine, DO doctor of osteopathic medicine, NP nurse practitioner, PA physician assistant, RN registered nurse

**Table 2:** Summary of the respondents’ experiences during the pandemic.

		N (%)
Does your local city/county/state have a mandatory mask order?	Yes	370 (91.8)
	No	27 (6.7)
	No answer	6 (1.5)
What % of local citizens are wearing masks in public?	< 25%	12 (3)
	26–50%	72 (17.9)
	51–75%	210 (52.1)
	>76%	108 (26.8)
	No answer	1 (0.2)
Have your local hospitals become overwhelmed with COVID-19 patients?	Yes	83 (20.6)
	No	319 (79.2)
	No answer	1 (0.2)
What is the approach the majority of schools are taking for return to school?	Temporary virtual learning, then transition to in-person	55 (13.6)
	Mix of virtual and in-person	225 (55.8)
	Virtual only	100 (24.8)
	In-person only	15 (3.7)
	Not sure	5 (1.2)
	No answer	3 (0.7)

**Fig 1:**
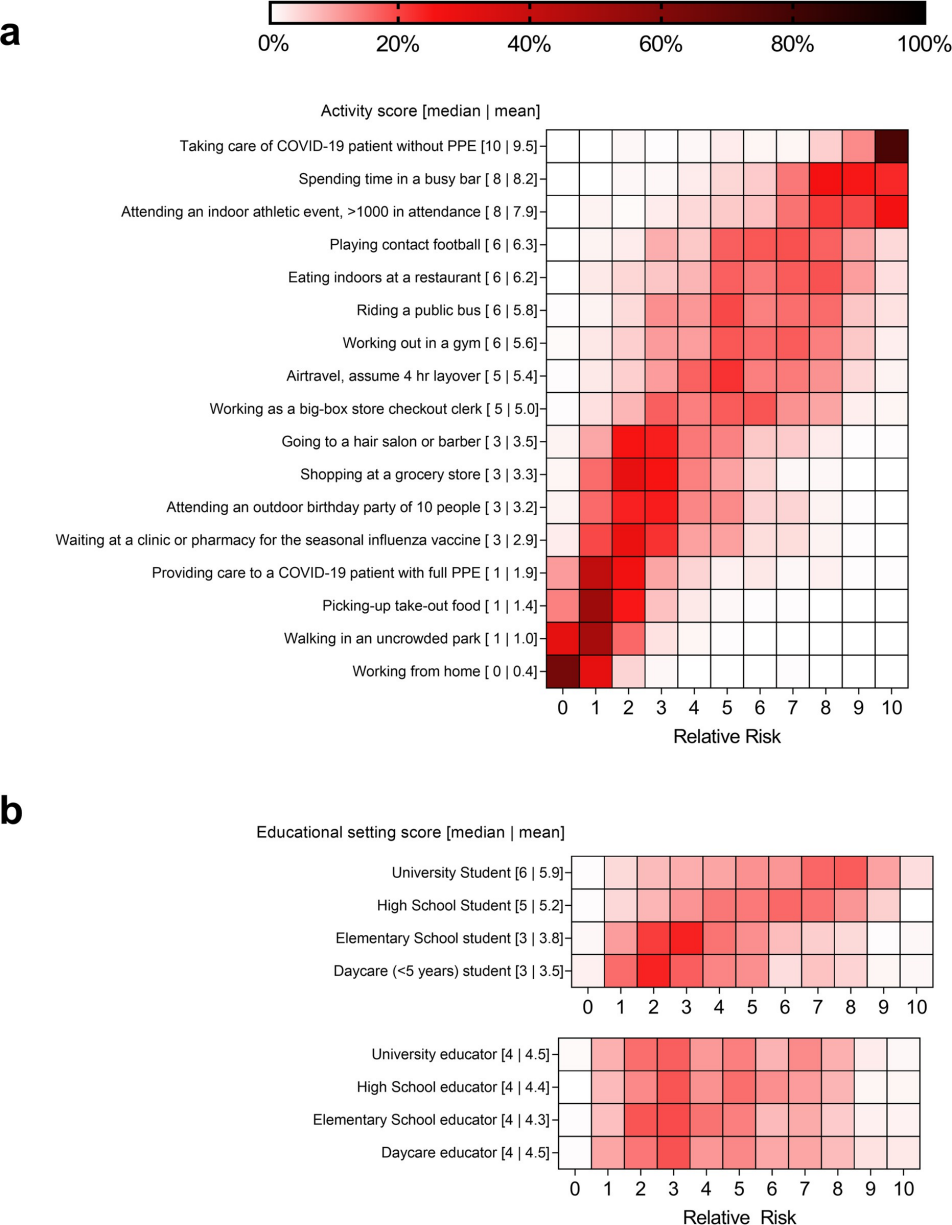
Heatmap of respondents risk assessment score. (a) social activities (b) educational setting. The frequency of responses for each score is represented by a percentage of the overall number of responses.

The activity associated with the highest risk was taking care of a COVID-19 patient without any PPE. Social activities that were perceived as the high risk of SARS-CoV-2 transmission prior to the availability of SARS-CoV-2 vaccines and the emergence of the delta variant included spending time in a bar and attending an indoor athletic event with >1000 in attendance (Fig 1A). Activities with the lowest risk scores include working from home, walking in an uncrowded park, picking up takeout food, and providing care to a COVID-19 patient with full PPE (Fig 1A). All other activities had greater disagreement in the risk assessment by respondents as >80% of scores ranged over 5 or more numerical values (Fig 1A). Respondents also estimated how the risk changed if no social distancing or mask wearing were implemented. On average for each respondent, the risk for each activity increased by 2.24 risk score units on a scale from 0 to 10, with the greatest increase for going to a hair salon or barber, shopping at a grocery store, and waiting at a clinic or pharmacy for the influenza vaccine.

We next determined the factors that influenced these risk assessments using the responses from US physicians with completed assessments for the activities. Both female gender (female = 6; males = 5.4; p value <0.001) and the absence of children in a household were associated with higher risk scores (no children = 6, with children = 5.5; p value = 0.008), when controlling for medical specialty, the time since training was completed, region of practice, local plans for return to school, if the respondent experienced a surge in COVID-19 cases, and the interaction of children in the household with time since finishing training (Table 3). All other predictors were non-significant (Table 3).

**Table 3:** Calculated marginal mean risk score and p values from the generalized linear model for predictors of risk of SARS-CoV-2 transmission in social activities.

Variable		Marginal mean risk score (95% CI)	P value
Gender	Male	5.4 (5.1–5.6)	**<0.001**
	Female	6.1 (5.8–6.3)	
Children (age <18 years) in the household	Yes	5.5 (5.2–5.8)	**0.007**
	No	5.9 (5.6–6.2)	
Have your local hospitals became overwhelmed with COVID-19 patients?	Yes	5.7 (5.2–5.8)	0.978
	No	5.7 (5.5–6.0)	
Specialty	Adult infectious diseases	5.6 (5.4–5.9)	0.216
	Pediatric Infectious diseases	5.6 (5.3–5.9)	
	General pediatrics	5.9 (5.5–6.3)	
Time in specialty	< 5 years	5.6 (5.3–6.0)	0.375
	5–14 years	5.9 (5.5–6.3)	
	15–24 years	5.8 (5.5–6.1)	
	>25 years	5.5 (5.1–5.9)	
Practice region	West	5.5 (5.1–5.9)	0.126
	Midwest	5.7 (5.4–6.0)	
	South	5.6 (5.3–6.0)	
	Northeast	6 (5.6–6.4)	
Schooling policy	Temporary virtual	5.9 (5.6–6.2)	0.297
	Mix of virtual or in-person	5.8 (5.6–6.1)	
	Virtual only	5.9 (5.6–6.2)	
	In-person only	5.2 (4.5–5.9)	

In the school setting, respondents identified the lowest risk of transmission of SARS-CoV-2 in daycare (age < 5 years; mean risk score 3.53) and elementary school (mean risk score 3.67) students compared to high school (mean risk score 5.2) or college (mean risk score 5.86; all p values ≤ 0.001; Fig 1B) level students. For teachers, no differences in the risk scores were identified between daycare (mean risk score 4.45), elementary (mean risk score 4.21), high school (mean risk score 4.43), or university (mean risk score 4.45; all p values ≥ 0.057). Respondents’ median and mean scores for SARS-CoV-2 transmission for elementary and daycare students were not significantly different when compared to getting a haircut at a salon or barber (adjusted p value = 1). For the perceived risk to college and high school students, the risk score was similar to the scores associated with riding a public bus, working out in a gym, or air travel (adjusted p value = 1). The median and mean risk scores attributed to university, high school, and daycare teachers were similar to scores found for working in a big box store as a checkout clerk (p values ≥0.408), while elementary school educators were statistically lower (p value = 0.019).

In a generalized linear model of the mean risk to students and teachers across all educational settings, we identified a lower risk score identified by general pediatricians (marginal mean risk score 4.49) and pediatric infectious diseases specialists (marginal mean risk score 4.57) compared to adult infectious diseases (marginal mean risk score 5.08; p = 0.046), and a difference amongst years since finished training (marginal mean risk score < 5 years: 4.9, 5–14 years: 5.21, 15–24 years: 4.69, >25 years 4.06 p = 0.037). To determine in what settings these predictors were significant, we used ordinal regression for each educational setting for educators and students. Pediatric infectious diseases physicians and general pediatricians rank the risk lower in elementary and daycare students and teachers compared to adult infectious diseases providers (Table 4), when controlling for gender, presence of children age <18 in the household, region of practice, if the respondent experienced a surge in COVID-19 cases, or local schooling policy. This association was not identified in the high school or university level for students or educators (Table 4).

**Table 4:** Adjusted odds ratio (95% confidence interval) of ordinal regression for predictors influencing the risk assessment for students and teachers in each educational environment.

		University student	High school student	Elementary student	Daycare student
Specialty	Adult infectious diseases	- (comparison)	- (comparison)	- (comparison)	- (comparison)
	Pediatric infectious diseases	1.02 (0.65–1.6)	0.95 (0.61–1.49)	**0.51 (0.32–0.8)** **	**0.4 (0.25–0.64)** ***
	General pediatrics	1.61 (0.85–3.06)	1.05 (0.55–1.99)	**0.27 (0.14–0.53)** ***	**0.16 (0.08–0.31)** ***
Gender	Male	- (comparison)	- (comparison)	- (comparison)	- (comparison)
	Female	0.96 (0.64–1.44)	1.15(0.76–1.74)	1.18 (0.78–1.79)	1.11 (0.73–1.68)
Children (age <18 years) in the household	Yes	- (comparison)	- (comparison)	- (comparison)	- (comparison)
	No	1.51 (0.91–2.52)	1.51(0.91–2.52)	1.21 (0.73–2.03)	1.16 (0.69–1.95)
Years since finished training	>25 years	- (comparison)	- (comparison)	- (comparison)	- (comparison)
	15–24	**1.91 (1.05–3.5)** *	**1.89 (1.03–3.46)** *	1.72 (0.94–3.17)	**1.9 (1.03–3.51)** *
	5–14	**3.83 (1.99–7.39)** ***	**2.51 (1.31–4.83)** **	1.2 (0.63–2.31)	1.45 (0.75–2.79)
	< 5	**2.05 (1.05–4.01)** *	**1.19 (1.05–4.01)** *	**2.5 (1.26–4.93)** **	1.69 (0.86–3.33)
Have your local hospitals became overwhelmed with COVID-19 patients?	Yes	- (comparison)	- (comparison)	- (comparison)	- (comparison)
	No	0.96 (0.57–1.63)	0.86 (0.51–1.46)	0.66 (0.38–1.12)	0.87 (0.51–1.48)
		University teacher	High school teacher	Elementary teacher	Daycare teacher
Specialty	Adult infectious diseases	- (comparison)	- (comparison)	- (comparison)	- (comparison)
	Pediatric infectious diseases	0.79 (0.5–1.24)	0.9 (0.58–1.41)	**0.52 (0.33–0.81)** **	**0.5 (0.32–0.8)** **
	General pediatrics	1.35 (0.71–2.57)	1.32 (0.69–2.5)	**0.45 (0.24–0.87)** *	**0.32 (0.17–0.62)** ***
Gender	Male	- (comparison)	- (comparison)	- (comparison)	- (comparison)
	Female	1.1 (0.72–1.66)	0.89 (0.59–1.34)	0.87 (0.57–1.32)	0.8 (0.53–1.21)
Children in home	Yes	- (comparison)	- (comparison)	- (comparison)	- (comparison)
	No	1.37 (0.82–2.29)	1.39 (0.83–2.32)	1.19 (0.72–1.99)	1.29 (0.77–2.15)
Years since finished training	>25 years	- (comparison)	- (comparison)	- (comparison)	- (comparison)
	15–24	**1.97 (1.07–3.61)** *	**2.08 (1.13–3.81)** *	1.33 (0.72–2.43)	1.51 (0.83–2.76)
	5–14	**3.5 (1.82–6.76)** ***	**2.87 (1.49–5.52)** **	1.47 (0.77–2.81)	1.33 (0.69–2.53)
	< 5	**2.69 (1.37–5.29)** **	**3.32 (1.68–6.54)** ***	**2.77 (1.41–5.46)** **	1.85 (0.94–3.63)
Have your local hospitals became overwhelmed with COVID-19 patients?	Yes	- (comparison)	- (comparison)	- (comparison)	- (comparison)
	No	0.64 (0.37–1.08)	**0.57 (0.33–0.97)** *	**0.46 (0.27–0.78)** **	0.6 (0.35–1.018)

* p value ≤0.05

** p value ≤0.01

*** p value ≤0.001

This study is the first assessment of general pediatricians’ and pediatric and adult infectious diseases specialists’ attitudes towards the risks of SARS-CoV-2 transmission for common activities prior to the availability of the SARS-CoV2 vaccines and emergence of the delta variant and quantification of the factors that influence these perceptions. Overall, the rankings mirror recommendations by which to reduce the likelihood of SARS-CoV-2 transmission, including maintaining low density of people, social distancing, and holding activities outdoors [26, 27]. Respondents identified activities with a high density of participants in an indoor space where masks are removed for eating and drinking as the highest risk activities, including spending time in a bar and attending an indoor sporting event. Many of the activities of the next tier of risk have features that maintain elevated risk, including being in close contact with many other members of the public [26, 27]. Interestingly, respondents ranked the risk of SARS-CoV-2 transmission higher for those working as a clerk at a big box store compared to providing care of COVID-19 patients with full PPE. This finding may reflect the interpretation that full PPE significantly reduces the likelihood of SARS-CoV-2 transmission beyond protective measures that could be implemented in public spaces. However, an increased risk of COVID-19 has been identified in healthcare workers compared to the general public [28, 29].

We also identified factors that influence these scores, notably that females assess the risk of SARS-CoV-2 transmission higher than males. This correlation has been found in other surveys assessing the risk of COVID-19 [17–19], other infectious diseases [20, 30, 31], and has been a recognized feature of risk analysis [32]. We also identified that respondents with children in their household rated the risks of SARS-CoV-2 transmission as less than those without children. There was no interaction of household children with years since finishing training, suggesting this finding is not due to different perceptions based on age of the respondents. Similar findings have been identified in regards to influenza risk assessment [20]. In contrast, surveys of residents of Chongqing City, China found that respondents with children at home ranked the risks of SARS-CoV-2 higher than those without children [19].

For risks of transmission in the school setting, respondents perceived that elementary and daycare students (age <5 years) were at the lowest risk for transmission of SARS-CoV-2 compared to high school or college students. This finding may reflect some of the preliminary data regarding reduced transmission of SARS-CoV-2 amongst younger children [8, 33]. It may also reflect the overall reduced incidence, morbidity, and mortality associated with COVID-19 in pediatric patients [34–36]. Our results also demonstrate that respondents identified greater risk for attending restaurants and bars compared to reopening high schools, elementary schools, or daycares for students (adjusted p value for all comparisons ≤ 0.001). Increased risk of transmission of SARS-CoV-2 has been previously associated with attending indoor restaurants and bars [6, 7].

Our assessment also identified that pediatric providers, including pediatric infectious diseases specialists, assess the risk of SARS-CoV-2 transmission in elementary or daycare students and teachers lower when compared to adult infectious disease specialists. These findings suggest that perceptions of risk could be influenced by the age group of their patient population. In addition, pediatric practitioners could be more significantly influenced by recommendations of national pediatric societies, as the American Academy of Pediatrics recommended school reopening for in-person instruction when possible [24]. In contrast, similar statements have not been made by major adult specialty societies. Pediatric practitioners could be more likely to understand other negative consequences that affect children’s well-being during the pandemic, as school closure has led to increased risk of food insecurity, child abuse, and concerns of increased depression and suicide [24].

Our study does have some limitations. We obtained responses from convenience samples of practitioners in infectious diseases and pediatrics. We did not send the assessment to medical providers in other medical specialties. It is possible that the risks of SARS-CoV-2 transmission could be differently interpreted by other specialties. The pediatric group who responded to our assessment reflects mostly practitioners in the St. Louis metropolitan area, and may not reflect attitudes of general pediatricians across the United States. In contrast, our respondents in adult and pediatric infectious diseases did represent all major regions of the United States. In the future, qualitative studies can be conducted to better understand the individual factors that influence risk perception by healthcare providers. This survey was also completed prior to the wide-spread availability of the SARS-CoV-2 vaccines and emergence of the delta variant. This assessment could be sent out again to determine how the attitudes change with time as the pandemic continues to evolve.

## Conclusions

Our survey provides an important summary of how approximately 400 healthcare providers interpret the risk of SARS-CoV-2 transmission in different activities and the educational setting. Our risk assessment rankings could be used to compare risks between activities, allowing for establishment of different risk tiers. These risk tiers can inform discussions of which groups of activities should be restarted first before higher risk activities resume.

## Supporting information

S1 Data Survey data. (CSV)
